# Meta-analyses in sport and exercise science: sometimes the right tool at the wrong time?

**DOI:** 10.1186/s13643-026-03148-3

**Published:** 2026-04-02

**Authors:** Sebastian Puschkasch-Möck, José Afonso, Konstantin Warneke, Klaus Wirth

**Affiliations:** 1 Department of Exercise Science, Olympic Training and Testing Center of Hessen, Frankfurt Am Main, Germany; 2 Centre of Research, Education, Innovation, and Intervention in Sport (CIFI2D), Faculty of Sport, University of Porto, Porto, Portugal; 3 Institute of Human Movement and Exercise Physiology, University of Jena, Jena, Germany; 4 Department of Sport Sciences, University of Applied Sciences Wiener Neustadt, Wiener Neustadt, Austria

**Keywords:** Clinical heterogeneity, Statistical heterogeneity, Study quality

## Abstract

**Background:**

Meta-analysis is widely regarded as a high-level form of evidence in scientific research, particularly in the medical and life sciences. In sport and exercise science, the number of published meta-analyses has increased rapidly in recent years. This expansion raises important questions about when quantitative synthesis is appropriate and when meta-analysis may become the right tool at the wrong time. While meta-analysis offers a powerful tool for synthesizing data from multiple studies, it is crucial to apply appropriate methodology to ensure the validity of results.

**Objective:**

The objective of this commentary is to clarify when meta-analysis is an appropriate method and when it risks providing misleading numerical precision, by critically discussing common methodological and interpretive pitfalls in sport and exercise science.

**Approach and results:**

It therefore examines common pitfalls in the execution and interpretation using exemplary meta-analyses published in sport and exercise science, focusing on issues such as clinical and statistical heterogeneity, the selection of appropriate statistical models, and the challenges posed by methodological differences across studies. Clinical heterogeneity, including variations in study populations, interventions, and outcomes, is particularly problematic and requires careful consideration when defining inclusion and exclusion criteria. Furthermore, statistical heterogeneity, which refers to the variability in effect sizes across studies, necessitates the use of random-effects models and sensitivity analyses to account for the diversity of included studies. The choice of statistical methods and the potential impact of outliers can significantly influence meta-analytic conclusions, highlighting the need for rigorous analytical approaches. This commentary also emphasizes that meta-analysis should not always be the default method for synthesizing evidence, particularly when studies are excessively heterogeneous or of low quality. Rather than blindly aggregating data, researchers must critically assess whether a meta-analysis is warranted and if the findings can be meaningfully interpreted.

**Conclusion:**

In conclusion, while meta-analysis is a valuable tool, its results should be carefully framed within the context of the studies included, acknowledging potential limitations and avoiding over-reliance on aggregated data.

## Background

Meta-analysis is often considered the highest level of scientific evidence [1] and is commonly advocated in medical and life science research. To facilitate transparent and complete reporting, the Preferred Reporting Items for Systematic reviews and Meta-Analyses (PRISMA) have been developed and later replaced by PRISMA 2020. While such tools improve how reviews are reported, they do not by themselves guarantee that appropriate methodological or analytical decisions have been made. Following these or similar guidelines like CASP [2] is usually necessary to publish a meta-analysis in scientific journals, but adherence primarily reflects reporting completeness rather than ensuring that the underlying synthesis methods are appropriate.

When analyzing the number of yearly meta-analyses published in PubMed registered journals since the year 2000, a nearly exponential increase can be observed. The question is whether there is enough new evidence to justify this exponential increase in meta-analyses. There is likely no general answer to this question. Some fields put out huge numbers of publications per year (e.g., studies on anterior cruciate ligament injuries), justifying the application of meta-statistics as a valuable instrument, while others may just have a couple of publications per year or be of such novelty that conducting a meta-analysis might be premature. Additionally, meta-analyses can focus on different aspects of a phenomenon, leading to multiple meta-analyses made upon the same original research, but tackling different aspects (e.g., meta-analyses on the effects of plyometric training). Finally, sometimes concepts are applied for decades in sports practice, but still have not been investigated. In such cases, approaches might be recommended assuming that there is evidence supporting their application. Under such circumstances, an attempted meta-analysis may end up being an empty review—but an important empty review nonetheless, as it will show that an entire concept or intervention has been supported for decades in the complete absence of research on the topic. Examples can be found in the work of Afonso et al. [3] on stretching as recovery from groin pain or injury and in the review by Weakley et al. [4], who found zero studies investigating overtraining syndrome in athletes.

First developed in 1976 by Gene V. Glass [5], the core aim of a meta-analysis is to synthesize the data of independent studies on a common topic to increase the sample size and to compute a combined effect size across these studies. While this is a generally interesting and favorable approach, leading to an exceptionally powerful tool if applied correctly and rigorously, caution and accuracy are required if the work is meant to generate meaningful added value. In the context of medication treatment studies, for example, the high methodological homogeneity enables meaningful calculation of meta-statistics, increasing the sample size drastically. In sport and exercise science, however, it has to be questioned whether sufficient comparability (e.g., participants, interventions, comparators, outcomes) between studies exists to use this tool appropriately.

According to Borenstein et al. [6], the procedure of conducting a meta-analysis begins with defining a clear research question, followed by an exhaustive literature search and selection of studies based on predefined inclusion and exclusion criteria. Data is extracted from eligible studies, including effect sizes and sample characteristics, while assessing study quality and risk of bias. A statistical model, typically a random-effects model, is used to combine effect sizes, with the impact of heterogeneity assessed through tests like the *I*^2^ or 95% prediction intervals, among other possibilities. The risk of publication bias is evaluated—although this is a contentious topic [7]—and sensitivity and subgroup analyses are conducted to explore potential sources of heterogeneity before interpreting and reporting the pooled results in the context of study limitations and practical significance. The way meta-analyses are conducted and interpreted has practical consequences. Overinterpretation of heterogeneous or weakly comparable studies may lead clinicians to adopt ineffective or even inappropriate interventions, mislead researchers in designing future trials, and ultimately affect the quality of care and decision-making experienced by patients.

The purpose of this commentary is to highlight some of the pitfalls when conducting and interpreting meta-analyses and to urge caution against over-reliance on the results of individual meta-analyses.

## Factors affecting the validity and interpretation of meta-analytic results

### Clinical heterogeneity: the need for clear inclusion and exclusion criteria

Clinical heterogeneity refers to variations across studies included in a meta-analysis concerning relevant characteristics [6]. This variability may manifest in differences in the studied populations (e.g., age groups, sex, training history, or health status), the types of interventions applied (e.g., different exercise modalities or dosages), the selected outcomes (e.g., maximal strength, isokinetic power, range of motion), or the measurement instruments used (e.g., differing methods for assessing muscular strength). High amounts of clinical heterogeneity can be a significant challenge in interpreting meta-analysis results, as it can undermine the comparability and generalizability of findings [8, 9]. To draw valid and meaningful conclusions, it is crucial to recognize and appropriately account for these differences (e.g., subgroup and/or sensitivity analyses) or decide not to pool the data at all [10].

One example that illustrates the importance of carefully selected inclusion criteria is the work of Nieto-Acevedo et al. [11], investigating sex differences in the mean propulsive velocities of resistance exercises at 30, 70, and 90% of the one-repetition maximum (1RM). Six studies were included in the analysis and revealed significant sex differences for 30% 1RM and 70% 1RM, but not 90% 1RM calculated via a random-effects model. A closer inspection reveals that five of the included studies investigated upper-body pushing movements like the military press or the bench press, while one study investigated a lower-body exercise, namely the squat. Considering the different results on velocity profiles for upper and lower-body exercises [12] as well as the difficulties when measuring movement velocities in different exercises [13], this combined analysis risks comparing apples to oranges. The single study investigating the squat showed exceptionally high mean values compared to the upper-body exercises, as well as higher effect sizes. When recalculating the standard mean differences (SMD) for upper-body exercises only, SMD values are 7.7–48.2% lower than the ones reported by Nieto-Acevedo et al. [11] (Table 1).

**Table 1:** Reported results and recalculation for the upper body of the meta-analysis by Nieto-Acevedo et al. [11]

Parameter	Nieto-Acevedo et al. (2023)				Recalculated for upper body only			
	**SMD**	**95% CI**	**sig**	**ES**	**SMD**	**95% CI**	**sig**	**ES**
30% 1RM	1.30	0.99–1.60	*p* < 0.00001	Large	1.20	0.86–1.55	*p* < 0.00001	Moderate
70% 1RM	0.92	0.63–1.21	*p* < 0.00001	Moderate	0.82	0.49–1.15	*p* < 0.00001	Moderate
90% 1RM	0.27	− 0.00–0.55	*p* = 0.05	-	0.14	− 0.18–0.45	*p* = 0.3924	-
Combined	0.99	0.63–1.35	*p* < 0.00001	Moderate	0.81	0.49–1.14	*p* < 0.00001	Moderate

*CI* confidence interval, *ES* effect size, *sig* statistical significance, *SMD* standard mean difference

This example illustrates the necessity for carefully chosen inclusion criteria to obtain meaningful results that can reliably be translated into scientific and training practice. Importantly, the inclusion criteria for the systematic review as a whole may not coincide with the inclusion criteria for entering the meta-analyses. Maybe several studies fulfill the review’s eligibility criteria but are sufficiently distinct from the other studies to preclude their inclusion in a meta-analysis. For example, if a review includes studies about stretching interventions, and a single study is performed with already injured subjects, this study probably requires a separate narrative analysis and will likely not enter meta-analytic calculations.

This problem compounds when comparing different meta-analyses on the same topic and therefore caution and attention to detail are warranted. A direct comparison is only justified when a similar or identical definition of terms is used. This can be illustrated by the works of Schoenfeld et al. [14] and Baz-Valle et al. [15] on the influence of resistance training volume on muscle hypertrophy, both using a three-level categorization of training volume as “low,” “medium,” and “high.” Schoenfeld et al. [14] observed a dose-response relationship between training volume and muscle hypertrophy with greater training volume producing greater hypertrophy. Baz-Valle et al. [15], however, did not observe significant differences between medium and high training volumes for the quadriceps and the biceps brachii. Conflicting at first glance, a look at the defined categories sheds light on this discrepancy. Schoenfeld et al. [14] defined their categories as < 5 weekly sets, 5–9 weekly sets, and 10 + weekly sets per muscle; Baz-Valle et al. [15] categorized the identified studies as < 12, 12–20, and > 20 weekly sets. All categories defined by Schoenfeld et al. [14] fall into the low volume category used by Baz-Valle et al. [15], and the results and conclusions of the two meta-analyses cannot be compared, even if the research question is identical. The research groups did not provide a rationale for the chosen cut-off values, highlighting the arbitrariness of both definitions.

Clinical heterogeneity often interacts with statistical heterogeneity, which refers to the variability in effect sizes across studies that cannot be attributed to sampling error alone [8]. While clinical heterogeneity addresses differences in study characteristics (such as population, intervention, and measurement methods), statistical heterogeneity is quantified by statistical measures, such as the popular *I*^2^ statistic (technically, this statistic addresses the *impact* of heterogeneity), that estimate the degree of inconsistency among study results. When studies exhibit significant clinical heterogeneity, it is more likely that statistical heterogeneity will also be observed, as the underlying differences in study design and execution can contribute to divergent effect sizes. Therefore, it is essential to assess both forms of heterogeneity in parallel to ensure the appropriateness of the meta-analytic model and the validity of the conclusions drawn.

### Statistical methodology: the importance of using appropriate analytical approaches

Statistical methodology plays a central role in meta-analysis, as it determines how data from multiple studies are combined to derive an overall estimate of effect [1, 8]. This is conceptually distinct from reporting quality. A review may be reported in full compliance with PRISMA while still relying on questionable analytical decisions, such as inappropriate pooling of heterogeneous studies. The primary challenge lies in addressing the variability across studies, which can arise from differences in study designs, populations, interventions, comparators, outcomes, and measurement methods. The choice of statistical model—whether fixed-effects or random-effects (which comes in different “flavors”)—influences the interpretation of the results. It is important to emphasize that the choice between fixed-effects and random-effects models is not merely a technical decision but reflects a conceptual choice about the target estimand. A fixed-effects model treats the included studies as the entire universe of interest and estimates a single common effect conditional on those studies. In contrast, a random-effects model assumes that true effects vary across studies and aims to describe the distribution of effects in a wider population of possible studies, not only those that happened to be included in the review. Consequently, random-effects models are not automatically “preferred” simply because statistical heterogeneity is present. Instead, researchers should first define what they intend to estimate: a common effect across highly similar studies, or the mean (and spread) of a distribution of genuinely different effects. When very few studies are available, random-effects models require particular caution, because between-study variance estimates are unstable and pooled estimates may be overly influenced by single studies [6, 10]. In such cases, prediction-oriented summaries, such as prediction intervals, may be more informative than focusing exclusively on the pooled average [10]. Moreover, substantial clinical heterogeneity may indicate that the studies should not be pooled at all, and that a narrative synthesis or stratified analyses are more appropriate than applying a random-effects model by default.

Furthermore, researchers must carefully consider factors such as heterogeneity and outliers, which can significantly affect the accuracy and reliability of meta-analytic conclusions [6]. A thorough understanding of these statistical principles is crucial to avoid erroneous interpretations and to ensure that the meta-analysis produces meaningful, evidence-based findings.

Another aspect to bear in mind is the impact of possible calculation errors, especially in meta-analyses including a small number of studies. Slimani et al. [16] performed a meta-analysis on lower-limb muscle power development in young athletes. For training-induced changes in squat jump (SJ) height, ten studies were included. When looking at the reported statistics for each study, one training group in the study of Lloyd et al. [17] stands out, showing the only negative value (SMD = −0.54) for the SMD. When going back to the original work of Lloyd et al. (2016) and comparing it with the metrics reported by Slimani et al. [16], it can be seen that the pre- and post-training values reported in the meta-analysis do not match the original study. When recalculating the SMD for this study, a value of 0.37 is obtained. Assuming that the SMDs for the other included studies are correct and using the new value for the work of Lloyd et al. [17], the reported random effect of SMD = 0.80 from Slimani et al. [16] needs to be corrected to SMD = 0.89.

In meta-analyses, choosing between fixed-effects and random-effects models is critical, as it impacts both results and interpretation [6]. A fixed-effects model assumes a consistent true effect size across all studies, with observed variation attributed to sampling error. It is appropriate for highly homogeneous studies in terms of population and intervention. In contrast, a random-effects model allows for variability in the true effect size across studies, accounting for both within-study and between-study variance. This model is often used when clinical or statistical heterogeneity is present, as it offers a more flexible and realistic estimate of the overall effect [8]. However, its use has been criticized. Greenland and O’Rourke [9] and Doi [8] point out that common study quality metrics are problematic. Researchers must carefully assess the level of heterogeneity to select the most appropriate model and draw valid conclusions.

Subgroup and sensitivity analyses are essential tools in meta-analysis to explore the robustness of the findings and to identify potential sources of heterogeneity [1]. Subgroup analysis involves dividing the data into smaller groups based on specific characteristics (e.g., age, sex, or intervention type) to investigate whether the effect size differs across these groups. Sensitivity analysis, on the other hand, assesses how changes in study inclusion criteria, model assumptions, or data handling affect the overall results [1]. For outliers as an example—a term without a single consensus definition—one might perform calculations with the outliers and then remove them to perform a sensitivity analysis. This allows assessing how much (if at all) they affect the overall results. These analyses help determine whether the conclusions of the meta-analysis are stable and generalizable or if they are overly influenced by specific studies or methodological choices. By conducting these analyses, researchers can enhance the transparency and reliability of their findings, ensuring that the conclusions drawn are not unduly affected by outliers or other biases.

Even with correctly calculated measures, the results of a meta-analysis should only be regarded as an interim summary of the existing body of knowledge on a topic rather than a permanent, irrefutable truth—especially if the methods of the included primary studies display considerable heterogeneity and/or are of low quality. An interesting example for this is the comparison of the two meta-analyses by Schoenfeld et al. [18] and Schoenfeld et al. [19] on the effects of volume-equated resistance training frequency on muscle hypertrophy. Both studies are authored by three people, of which two are identical, as were two of the three used databases each. Using comparable inclusion criteria, the earlier work included ten studies into the meta-analysis while the later work identified 25 studies that met the criteria. While the earlier analysis resulted in a recommendation of 2 + resistance training sessions per week, the later analysis incorporating a greater number of studies could not identify a significant effect of resistance training frequency when training volume is equated. This illustrates several problems when interpreting the results of meta-analyses. The newer meta-analysis contradicting the older one does not justify an interpretation of one of the results as reflecting the truth more than the other. Possibly, another meta-analysis a few years from now, collecting even more studies on the topic, might shift the recommendation toward a higher training frequency again. A training study justifying its loading protocol on the results of Schoenfeld et al. [18] might incorporate a completely different design than a study based on Schoenfeld et al. [19]. Therefore, study results—and possibly future meta-analyses on the investigated parameters—might or might not be influenced by training frequency.

Besides different definitions of used categories and terms, the incorporation or neglect of influencing factors (e.g., cointerventions, nuisance variables) has to be regarded as highly influential on the results of a meta-analysis. While Schoenfeld et al. [14] did not control for the muscles investigated and calculated an overall effect only, Baz-Valle et al. [15] differentiated their analysis according to the muscle groups investigated and found a different effect for the triceps brachii than for the quadriceps or the biceps brachii. When looking at the training duration of the studies incorporated by Baz-Valle et al. [15], one study stands out at 24 weeks compared to all other studies ranging between 6 and 8 weeks of training. This longer study had by far the highest effect favoring high training volume over medium training volume. The selection of which factors should be considered, and which should be ignored, is subjective to a certain extent. Additional factors like measurement procedures have to be regarded as influential on the study outcomes as well. When conducting measurements on muscular adaptations to training, for example, it has to be highlighted that muscle thickness, muscle cross-sectional area, and muscle volume display measures of different dimensions [20] with very distinct measurement errors and reliabilities [21].

## Is aggregating data always the right answer?

Caution is warranted when interpreting a meta-analysis covering a wide range of possibly positive and negative effects and calculating a mean effect size deemed as meaningful. The usefulness of this approach relies on methodological appropriateness and rigor, especially with respect to moderating or confounding variables. While the systematic review and report of existing studies on a common topic may be useful, the outcomes of this qualitative, narrative synthesis should determine whether a meta-analysis should be calculated or not. After all, even if meta-analytical procedures were planned, researchers are free to decide against following through if they deem the included studies excessively heterogeneous [10]. Even if meta-analyses are performed, perhaps the inclusion criteria were not refined enough and/or covariates differed between the studies, influencing the results.

Therefore, depending on the context and on the pondering of several factors, a meta-analysis might be the right tool at the wrong time. The aggregated overview of heterogeneous results has to be regarded as a critical step to point out limitations in established theories and to search for factors explaining the contradictory findings. However, excessive heterogeneity and poorly defined eligibility criteria mean the results of any meta-analysis must be framed within the appropriate context. In a nutshell, the results must not be taken at face value. The number and quality of identified studies should also influence whether a systematic review will follow through with preplanned meta-analyses. An example is the meta-analysis on the influence of strength training on motor unit discharge rates by Elgueta-Cancino et al. [22], which retrieved seven studies that met the inclusion criteria, and not a single study was rated with a low risk. For the calculation, only four studies were included, with participant numbers per group ranging between 8 and 13. The authors highlighted the lack of high-quality evidence on the topic and that the results of their meta-analysis (no significant main effect) should be interpreted with caution. The systematic reporting of low numbers and quality of studies in the field may be highly valuable and informative, but meta-analyses may render meaningless results if interpreted without considering their full context.

To support transparent and defensible decisions about whether quantitative pooling is appropriate, we propose the following practical decision framework. Before undertaking a meta-analysis, authors should first ensure that the target construct is clearly and consistently defined across studies. Potential sources of effect heterogeneity—such as measurement method, anatomical region, exercise modality, or intervention duration—should be identified a priori and evaluated as plausible effect modifiers. Where meaningful differences between studies exist, pooling should be restricted to theoretically and methodologically defensible strata rather than across fundamentally distinct contexts. Studies that cannot be rendered comparable without strong assumptions should be synthesized narratively instead of being forced into a statistical model. Applying this framework may prevent the inappropriate use of meta-analysis in situations where heterogeneity undermines the interpretability of pooled estimates. To facilitate transparent and defensible decisions about meta-analytic pooling, these considerations are summarized in a practical decision framework (Table 2).

**Table 2:** A decision framework for responsible use of meta-analysis in sport and exercise science

Decision domain	Key question	Examples/indicators	Recommended approach
Target construct	Is the construct clearly and consistently defined across studies?	Divergent operational definitions; conceptually overlapping but non-identical outcomes	Do not pool unless construct equivalence can be justified
Effect modifiers	Are plausible sources of heterogeneity identifiable a priori?	Measurement method, anatomical region, exercise modality, intervention duration	Assess modifiers explicitly; consider stratified analyses
Contextual comparability	Are studies sufficiently similar in theoretical and methodological context?	Acute vs. chronic interventions; different training paradigms	Restrict pooling to defensible strata
Statistical justification	Does pooling meaningfully improve inference rather than numerical precision?	High heterogeneity with few studies; unstable estimates	Avoid meta-analysis if interpretability is compromised
Alternative synthesis	Can non-comparable studies be synthesized without pooling?	Conceptually relevant but methodologically distinct studies	Prefer structured narrative synthesis

## Conclusions

In summary, meta-analysis remains a powerful tool for evidence synthesis, but its utility in sport and exercise science is frequently constrained by substantial heterogeneity, small numbers of available studies, and imprecisely defined constructs. Under such conditions, pooled estimates may create an illusion of numerical precision without yielding meaningful scientific inference. Consequently, systematic reviews should not default to quantitative synthesis. In some cases, the most rigorous and informative conclusion is that the existing evidence base is too heterogeneous to justify meta-analysis at the present time.

## Data Availability

Not applicable.
